# Vitiligo: a call for paradigm shift toward comprehensive patient care

**DOI:** 10.3389/fmed.2025.1504460

**Published:** 2025-02-20

**Authors:** Julia Sigova, Maria Borodina, Aliya Kassymkhanova, Nigora Murotova, Konstantin Lomonosov, Torello Lotti

**Affiliations:** ^1^Department of Neonatology, Faculty of Continued Medical Education, Pirogov Russian National Research Medical University, Moscow, Russia; ^2^Department of Dermatology, University of Studies Guglielmo Marconi, Rome, Italy; ^3^Russian Children's Clinical Hospital, Pirogov Russian National Research Medical University, Moscow, Russia; ^4^Vitiligo Center, Almaty, Kazakhstan; ^5^Center for the Development of Professional Qualifications of Medical Workers, Department of Medical Engineers and Technicians in Medical and Innovative Technologies, Tashkent, Uzbekistan; ^6^Department of Dermatology and Communicable Diseases, Ministry of Health, I.M. Sechenov First Moscow State Medical University, Moscow, Russia

**Keywords:** quality of life, psychosocial, vitiligo, artificial intelligence, psychiatric comorbidity

## 1 Introduction

Vitiligo affects millions globally, yet care remains fragmented and insufficiently holistic. This work builds on the EADV Task Force's position on quality of life (QoL), proposing a paradigm shift that redefines vitiligo as a complex condition with physical, psychological, and social dimensions (1). Beyond dermatologic concerns, this approach integrates emerging tools like artificial intelligence (AI) and emphasizes comprehensive care to improve patient outcomes.

## 2 Real-world considerations in vitiligo

### 2.1 Prevalence and impact

Vitiligo affects 0.5%–1.5% of the global population, disproportionately impacting youth and working-age adults (2). Societal biases against skin variations exacerbate the psychological and social challenges faced by individuals, particularly in regions where skin color holds significant cultural importance.

### 2.2 Triggers and aggravation factors

Vitiligo is complex, with multiple potential triggers and aggravating factors that may persist even after successful treatment. These include physical trauma (Koebner phenomenon), emotional stress, hormonal changes, and exposure to certain chemicals or medications. Recent metagenomic sequencing study demonstrates a credible basis for exploring altered gut microbiota in patients with non-segmental vitiligo (3, 4). In some Central Asian countries, like Kazakhstan and Uzbekistan, hospital intake screening often reveals gut dysbiosis and parasitic infestations in vitiligo patients, emphasizing the need for local research and tailored interventions.

### 2.3 Psychiatric comorbidities

The prevalence of psychiatric comorbidities among vitiligo patients is alarmingly high. Studies report a wide range of prevalence for depression (0.1%−62.3%) and anxiety (1.9%−67.9%) (5). Vitiligo patients also have a significantly higher risk of obsessive-compulsive disorder, adjustment disorders, social phobia, and sleep disturbances (6). Suicidal ideation is a significant concern, particularly in children and adolescents with vitiligo.

In line with these observations, we propose a detailed psychological screening protocol. This includes administering validated tools such as the Patient Health Questionnaire-9 (PHQ-9) for depression and the Generalized Anxiety Disorder-7 (GAD-7) for anxiety. Additionally, referrals to mental health professionals should be routine for patients exhibiting signs of psychiatric distress.

### 2.4 Physiological impact beyond the skin

Vitiligo's physiological impact extends beyond the skin, with links to visual and auditory abnormalities, and heart conditions (7). The role of resident memory T cells (TRM) emphasizes the connection between vitiligo and the nervous system (8). This interaction may contribute to the pathogenesis of organ-specific autoimmune disorders often associated with vitiligo, such as inflammatory bowel disease, multiple sclerosis, and type 1 diabetes mellitus.

### 2.5 Psychosocial implications

Vitiligo significantly impacts patients' emotional wellbeing and social interactions (9). Many individuals experience stress, anxiety, depression, and low self-esteem, often feeling self-conscious about their appearance. Social stigma further exacerbates these challenges, as patients may face ostracism, teasing, and misconceptions about the disease being contagious. This stigma can affect personal relationships, career opportunities, and marriage prospects, particularly in communities where skin conditions carry strong social stigma.

### 2.6 Economic and familial considerations

Assessing both healthcare and non-healthcare costs associated with vitiligo is crucial, particularly in countries lacking government or commercial insurance support (9). The impact on parents' mental health, quality of life, and socioeconomic status must also be considered, especially in developing countries where cultural and social pressures exacerbate these challenges.

## 3 Artificial intelligence in vitiligo management

AI holds significant promise for managing vitiligo, especially in developing countries with limited access to advanced treatments and basic phototherapy tools (10). However, current AI diagnostic systems struggle due to a lack of diverse image datasets, particularly for populations with skin of color (SOC) (11).

A major limitation is regional bias in training datasets. The commonly used Fitzpatrick Skin Phototype scale fails to fully capture the range of SOC tones, especially in regions like Central Asia. Addressing this requires collaboration with local healthcare providers and the integration of more inclusive classifications, such as the Monk Skin Tone Scale, to improve diagnostic accuracy.

Other challenges include the high cost of AI integration and technological gaps in underserved areas. Partnerships with non-profits and government programs can help overcome these barriers by subsidizing deployment and training healthcare workers in AI-assisted care.

A notable example of an AI application in resource-limited settings is the AI-Guide on Vitiligo, developed by the non-profit Vitiligo Research Foundation (VRF) (12). Acting as a prototype for future teledermatology and patient support services, this human-like AI avatar now provides real time responses via text, voice, or video. It expertly covers topics from triggers to mental health, explores a full range of treatment options, and connects patients with nearby specialists.

## 4 Proposed comprehensive care approach

We propose reframing vitiligo as a condition requiring comprehensive care, aligning with the World Health Organization's definition of health as encompassing physical, mental, and social wellbeing. This holistic approach involves the following five key components:

1. Routine psychological screening: conducting regular assessments for psychological distress and psychiatric comorbidities in vitiligo patients using tools like PHQ-9 and GAD-7.
2. Multidisciplinary care teams: establishing integrated teams of dermatologists, mental health professionals, and social workers to address comprehensive patient needs.
3. Expanded research focus: prioritizing research into treatments that address both skin repigmentation and psychosocial wellbeing.
4. Enhanced healthcare provider education: training on the psychological impacts of vitiligo, supported by studies demonstrating the benefits of empathy-driven care.
5. AI-driven tools integration: leveraging advanced AI tools for precise diagnostics and tailored treatment pathways. Developing AI algorithms based on the Monk rather than Fitzpatrick scale, which better accounts for diverse skin color and ethnic background.

This approach would offer more accurate, culturally sensitive diagnostics and treatment pathways, particularly benefiting populations with skin of color.

## 5 Discussion

Vitiligo is a multifaceted condition requiring a holistic approach that integrates physical, psychological, and systemic factors. Addressing gut health, psychiatric comorbidities, and societal stigma is vital for improving QoL. AI offers promise in enhancing care but must be implemented equitably, with attention to data inclusivity and regional representation, to avoid gaps in AI training datasets and algorithms.

This paradigm shift underscores the importance of patient-centered, multidisciplinary care in transforming vitiligo management and achieving meaningful improvements in QoL for affected individuals.

## References

[1] Chernyshov PV Tomas-Aragones L Manolache L Pustisek N Salavastru CM Marron SE. Quality of life measurement in vitiligo. Position statement of the European academy of dermatology and venereology task force on quality of life and patient oriented outcomes with external experts. J Eur Acad Dermatol Venereol. (2023) 37:21–31. 10.1111/jdv.18593 36259656

[2] Spritz RA Santorico SA. The genetic basis of vitiligo. J Invest Dermatol. (2021) 141:265–73. 10.1016/j.jid.2020.06.004 32778407

[3] Al-Smadi K Leite-Silva VR Filho NA Lopes PS Mohammed Y. Innovative approaches for maintaining and enhancing skin health and managing skin diseases through microbiome-targeted strategies. Antibiotics (Basel). (2023) 12:1698. 10.3390/antibiotics12121698 38136732 PMC10741029

[4] Luan M Niu M Yang P Han D Zhang Y Li W. Metagenomic sequencing reveals altered gut microbial compositions and gene functions in patients with non-segmental vitiligo. BMC Microbiol. (2023) 23:265. 10.1186/s12866-023-03020-7 37737154 PMC10515041

[5] Ezzedine K Eleftheriadou V Jones H Bibeau K Kuo FI Sturm D. Psychosocial effects of vitiligo: a systematic literature review. Am J Clin Dermatol. (2021) 22:757–74. 10.1007/s40257-021-00631-6 34554406 PMC8566637

[6] Lai YC Yew YW Kennedy C Schwartz RA. Vitiligo and depression: a systematic review and meta-analysis of observational studies. Br J Dermatol. (2017) 177:708–18. 10.1111/bjd.15199 27878819

[7] Hu Z Wang T. Beyond skin white spots: vitiligo and associated comorbidities. Front Med. (2023) 10:1072837. 10.3389/fmed.2023.1072837 36910477 PMC9995999

[8] Ryan GE Harris JE Richmond JM. Resident memory T cells in autoimmune skin diseases. Front Immunol. (2021) 12:652191. 10.3389/fimmu.2021.652191 34012438 PMC8128248

[9] Mahama AN Haller CN Ahmed AM. Psychosocial considerations in the management of vitiligo. Clin Dermatol. (2023) 41:82–8. 10.1016/j.clindermatol.2023.02.008 36878455

[10] Hillmer D Merhi R Boniface K Taieb A Barnetche T Seneschal J. Evaluation of facial vitiligo severity with a mixed clinical and artificial intelligence approach. J Invest Dermatol. (2024) 144:351–357.e4. 10.1016/j.jid.2023.07.014 37586608

[11] Guo L Yang Y Ding H Zheng H Yang H Xie J. deep learning-based hybrid artificial intelligence model for the detection and severity assessment of vitiligo lesions. Ann Transl Med. (2022) 10:590. 10.21037/atm-22-1738 35722422 PMC9201159

[12] AI-Guide On Vitiligo. Available at: https://vitiligo.ai (accessed January 29, 2025).

